# Antinociceptive Activity of Methanol Extract of *Muntingia calabura* Leaves and the Mechanisms of Action Involved

**DOI:** 10.1155/2012/890361

**Published:** 2012-04-26

**Authors:** M. H. Mohd. Sani, Z. A. Zakaria, T. Balan, L. K. Teh, M. Z. Salleh

**Affiliations:** ^1^Department of Biomedical Sciences, Faculty of Medicine and Health Science, Universiti Putra Malaysia, Selangor, 43400 Serdang, Malaysia; ^2^Pharmacogenomics Center, Faculty of Pharmacy, Universiti Teknologi MARA, Selangor, 42300 Puncak Alam, Malaysia

## Abstract

*Muntingia calabura* L. (family Elaeocarpaceae) has been traditionally used to relieve various pain-related ailments. The present study aimed to determine the antinociceptive activity of methanol extract of *M. calabura* leaves (MEMC) and to elucidate the possible mechanism of antinociception involved. The *in vivo* chemicals (acetic acid-induced abdominal constriction and formalin-, capsaicin-, glutamate-, serotonin-induced paw licking test) and thermal (hot plate test) models of nociception were used to evaluate the extract antinociceptive activity. The extract (100, 250, and 500 mg/kg) was administered orally 60 min prior to subjection to the respective test. The results obtained demonstrated that MEMC produced significant (*P* < 0.05) antinociceptive response in all the chemical- and thermal-induced nociception models, which was reversed after pretreatment with 5 mg/kg naloxone, a non-selective opioid antagonist. Furthermore, pretreatment with L-arginine (a nitric oxide (NO) donor), N^G^-nitro-L-arginine methyl esters (L-NAME; an inhibitor of NO synthase (NOS)), methylene blue (MB; an inhibitor of cyclic-guanosine monophosphate (cGMP) pathway), or their combination also caused significant (*P* < 0.05) change in the intensity of the MEMC antinociception. In conclusion, the MEMC antinociceptive activity involves activation of the peripheral and central mechanisms, and modulation via, partly, the opioid receptors and NO/cGMP pathway.

## 1. Introduction

*Muntingia calabura *L. (family Elaeocarpaceae), the sole species in the genus Muntingia, is a flowering plant native to southern Mexico, the Caribbean, Central America, and western South America. The tree grows very easily and is widespread, and, in Malaysia, it is popularly known as “*Kerukup Siam*.” Despite less attention given to its medicinal values in the Malay folklore medicine, *M. calabura* has been traditionally used by the Peruvian to treat various ailments [1, 2]. According to the Peruvian folklore, its leaves can either be boiled or steeped in water to provide relief from gastric ulcer or to reduce swelling of the prostate gland, while the strips of its bark are boiled and washed to reduce the swelling in the lower extremities [3]. The leaves, in particular, have been used to treat pain associated with gastric ulcers, headache, and cold or to attenuate the prostate gland swelling [1, 2, 4]. Scientifically, the leaves of *M. calabura *have been reported to possess antitumour [5, 6], antinociceptive [3, 7, 8], anti-inflammatory and antipyretic [3, 9], antibacterial [10], and antiproliferative and antioxidant [11] activities of *M. calabura *leaves. Phytochemical screening of the leaves demonstrated the presence of flavonoids, saponins, tannins, triterpenes, and steroids, but no alkaloids [8], while the phytochemical analysis of methanol extract of *M. calabura* leaves (MEMC) revealed only the presence of flavonoids, saponins, and tannins [11]. The previous antinociceptive, anti-inflammatory, and antipyretic activity of *M. calabura* leaves, in particular, has been investigated using the aqueous extract of the leaves. Thus, the present study aims to report for the first time the antinociceptive profile of MEMC and the possible mechanism of actions involved.

## 2. Methodology

### 2.1. Plant Collection

The leaves of *M. calabura*, collected from its natural habitat in Shah Alam, Selangor, Malaysia, were reidentified by Mr. Shamsul Khamis from the Institute of Bioscience (IBS), Universiti Putra Malaysia (UPM), Serdang, Selangor, Malaysia. A voucher specimen (SK 1095/05) has been deposited at the Herbarium of the Laboratory of Natural Products, IBS, UPM, Serdang, Selangor, Malaysia.

### 2.2. Preparation of MEMC

This procedure was carried out as described in detail by Zakaria et al. [11]. Briefly, 500 g of matured leaves that have been air-dried for 1-2 weeks at room temperature (27 ± 2°C) and grinded into powder were soaked in methanol in the ratio of 1 : 20 (w/v) for 72 hours. After that, the supernatant was filtered using steel filter, cotton, and Whatman no. 1 filter paper. The residue was subjected to the same procedures for another two times. The supernatant collected from each extraction was pooled together and then subjected to evaporation process using a rotary evaporator at 40°C under reduced pressure.

### 2.3. Drugs and Chemicals

The following reagents and drugs were used: methanol (Fischer Scientific, UK), DMSO, formalin, acetic acid, morphine, acetylsalicylic acid (ASA), naloxone, capsaicin, glutamate, L-arginine, N^G^-nitro-L-arginine methyl esters (L-NAME), and methylene blue (MB) (Sigma, USA). The drugs were prepared by dissolving them into distilled water. The MEMC was dissolved in vehicle (10% DMSO) just before used. All solutions were administered in the volume of 10 mL/kg.

### 2.4. Animals

Male Sprague Dawley rats (180–200 g; 8–10 weeks old) and male ICR mice (25–30 g; 5–7 weeks old) obtained from the Veterinary Animal Unit, Faculty of Veterinary Medicine, Universiti Putra Malaysia (UPM), Malaysia, and kept under room temperature (27 ± 2°C; 70–80% humidity; 12 h light/darkness cycle) in the Animal Holding Unit (UPM), were supplied with food and water *ad libitum* up to the beginning of the experiments. The rats were, at all times, handled in accordance with current UPM guidelines for the care of laboratory animals and the ethical guidelines for investigations of experimental pain in conscious animals [12]. All experiments (*n* = 6) were conducted between 09.30 and 18.30 h to minimize the effects of environmental changes.

### 2.5. Antinociceptive Activity

#### 2.5.1. Acetic-Acid-Induced Abdominal Constriction Test

The acetic-acid-induced abdominal constriction test was carried out according to the method described by Zakaria et al. [3] with slight modification. The mice (*n* = 6) were pretreated with 10% DMSO (negative control), 100 mg/kg ASA (positive control), or MEMC (100, 250, and 500 mg/kg). Sixty minutes after the respective test solution administration, the mice were injected via intraperitoneal (i.p.) route with phlogistic agent (0.6% acetic acid). The animals were immediately placed individually into glass cage, and 5 min were allowed to elapse. The abdominal constriction resulting from the injection of acetic acid consists of a contraction of the abdominal together with a stretching of at least one hind limb. The number of abdominal constrictions produced in these animals was counted cumulatively for 25 min. Antinociceptive activity, indicated by the reduction in the mean of the number of abdominal constrictions in the test groups compared to the control group, was calculated as the percentage inhibition of abdominal constrictions (percentage of inhibitory level) using the following formula: (mean of (control-test group)/control group × 100%).

#### 2.5.2. Hot Plate Test

The hot plate test was carried out according to the method described by Wilson et al. [13] with some modifications. The temperature of the metal surface (Ugo Basile 7280) was set at  50 ± 0.2  °C. The mice (*n* = 6) were pretreated with 10% DMSO (negative control), 5 mg/kg morphine (positive control), or MEMC (100, 250, and 500 mg/kg). Sixty minutes after the respective test solution administration, the mice were placed on the heated metal surface and the latency to a discomfort reaction (licking paws or jumping) was recorded. The cut-off time of 20 s was chosen to avoid tissue injury. Latency was record before and 60, 90, 120, 150, 180, 210 min following oral administration of the treatments. The prolongation of the latency times compared with the values of the controls was used for statistical comparison.

#### 2.5.3. Formalin-Induced Paw Licking Test

The formalin test was carried out as described by Zakaria et al. [3] but with slight modifications. Pain was induced by injecting 50 *μ*L of 5% formalin in the subplantar region of the right hind paw. Rats (*n* = 6) were orally administered with 10% DMSO (negative control), 100 mg/kg ASA, 5 mg/kg morphine (positive control), or MEMC (100, 250, and 500 mg/kg) 60 min prior to the formalin injection. Immediately after the phlogistic agent administration, the rats were individually placed in a transparent glass cage observation chamber. The amount of time that the animal spent licking the injected paw, considered as an indicator of pain, was recorded for duration of 30 min in two phases, known as the early (0–5 min) and late (15–30 min) phases.

#### 2.5.4. Capsaicin-Induced Paw Licking Test

To investigate the role of vanilloid receptors in the modulation of MEMC antinociceptive action, the procedure described by Goncales et al. [14] was adopted with slight modifications. Rats were pretreated orally with 10% DMSO or MEMC (100, 250, and 500 mg/kg) 60 min before capsaicin injection (1.6 ug/paw, 20 uL) into the intraplantar (i.pl.) region of the rat's right hind paw. Immediately after the phlogistic agent administration, the rats were individually placed in a transparent glass cage observation chamber and observed individually for 5 min after the capsaicin injection. The amount of time the animals spent licking the injected paw was recorded with a chronometer and was considered as an indication of nociception.

#### 2.5.5. Glutamate-Induced Paw Licking Test

To study the role of glutamatergic system in the modulation of MEMC antinociceptive action, the procedure described by Beirith et al. [15] with slight modifications were performed. Rats were pretreated orally with 10% DMSO or MEMC (100, 250, and 500 mg/kg) 60 min prior to glutamate injection. A volume of 20 *μ*L of glutamate (10 umol/paw, in normal saline) was injected via i.pl. route in the right hind paw of rats. Immediately after the phlogistic agent administration, the rats were individually placed in a transparent glass cage observation chamber and observed individually from 0 to 15 min after the glutamate injection. The amount of time the animals spent licking or biting the injected paw was recorded with a chronometer and was considered as an indication of nociception.

#### 2.5.6. Involvement of Opioid Receptor

To determine the role of opioid receptors in the modulation of MEMC antinociceptive activity, a separate procedure described by Zakaria et al. [16] was adopted with slight modifications. Two groups of animals (*n* = 6) were pretreated (i.p.) with a nonselective opioid antagonist, naloxone (5 mg/kg; i.p) for 15 min followed by the oral administration of the most effective MEMC dose (500 mg/kg). Sixty minutes later, the animals are subjected to the acetic-acid-induced abdominal writhing test and the formalin test.

#### 2.5.7. Involvement of Nitric Oxide/Cyclic-Guanosine Monophosphate Pathway

To determine the role of nitric oxide/cyclic-guanosine monophosphate (NO/cGMP) pathway in the modulation of MEMC antinociceptive activity, the method described by Zakaria et al. [16] was adopted with slight modifications. Mice (*n* = 6) were pretreated with 20 mg/kg L-arginine, L-NAME, MB, or their respective combination (L-arginine with L-NAME or L-arginine with MB) followed 5 min later by pretreatment with 10% DMSO or MEMC (500 mg/kg), respectively. Sixty minutes after the administration of test solutions, the mice were injected (i.p.) with 0.6% acetic acid.

### 2.6. Statistical Analysis

The results are presented as Mean ± standard error of mean (SEM). The one-way ANOVA test with Dunnett post hoc test was used to analyze and compare the data, with *P* < 0.05 as the limit of significance.

## 3. Result

### 3.1. Acetic-Acid-Induced Abdominal Constriction Test

**Figure 1:**
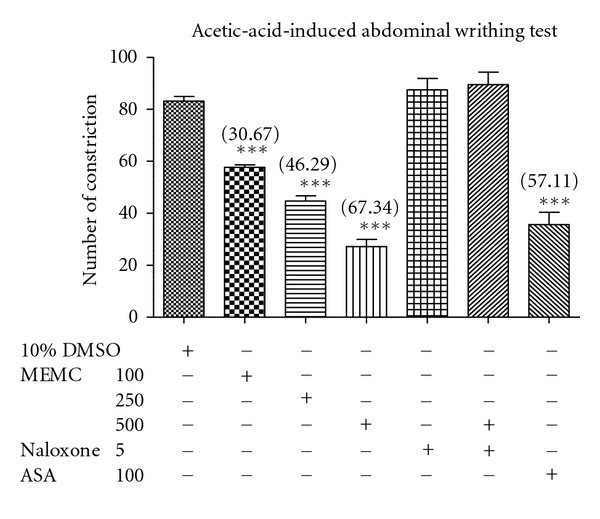
Effect of MEMC in acetic-acid-induced abdominal constriction test in mice. Acetic acid administered intraperitoneally 60 min before pretreated with vehicle (control), acetylsalicylic acid (ASA), or MEMC (100, 250, and 500 mg/kg). All treatments administered via oral route. The asterisks denote the significance levels as compared to control, ***P < 0.001 by one-way ANOVA followed by Dunnett's post hoc test. ***Data differed significantly (*P* < 0.001) when compared to the 10% DMSO-treated group.

The MEMC (100, 250, and 500 mg/kg, p.o.) demonstrated a significant (*P* < 0.001) and dose-dependent antinociceptive activity in the acetic-acid-induced abdominal constriction test (Figure 1) with the percentage of analgesia ranging between 30 to 67%. The 250 mg/kg MEMC produced an antinociceptive activity that was comparable to the standard control (100 mg/kg ASA).

### 3.2. Hot Plate Test

Table 1 shows the antinociceptive profile of orally administered MEMC assessed using the hot plate test. MEMC, only at the dose of 500 mg/kg, exhibited significant (*P* < 0.001) ability to prolong the latency of response to discomfort against thermal-induced nociception throughout the whole experiment. Overall, 5 mg/kg morphine demonstrated the most effective effect when compared to the MEMC at all doses used.

### 3.3. Formalin-Induced Paw Licking Test

**Figure 2:**
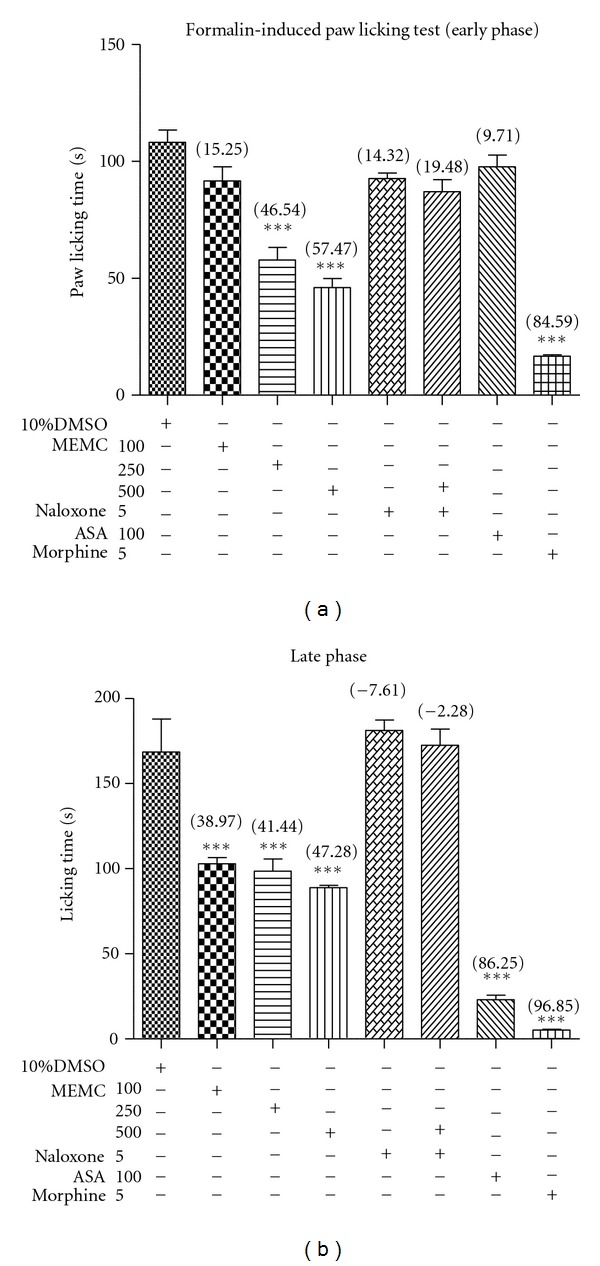
Effect of MEMC in formalin-induced paw licking test. Graph A shows early phase activity, while graph B shows the late phase analgesic effect. Each column represents the mean ± SEM of 6 rats. The rats were pretreated with vehicle (10% DMSO), MEMC (100, 250, and 500 mg/kg, p.o.), acetylsalicylic acid (ASA, p.o.), or morphine (5 mg/kg, p.o.), 60 min before i.pl injection of formalin. The asterisks denote the significance levels as compared to control, ***P < 0.001 by one-way ANOVA followed by Dunnett's post hoc test. ***Data differed significantly (*P* < 0.05) when compared to the 10% DMSO-treated group.

Overall, the MEMC demonstrated a significant (*P* < 0.05) antinociceptive activity in both phases of the formalin-induced paw licking test (Figures 2(a) and 2(b)). The extract exhibited a dose-dependent effect in the early, but not late, phase with antinociceptive activity seen only with the 250 and 500 mg/kg MEMC, whereas, in the late phase, all doses of MEMC exerted significant (*P* < 0.05) antinociceptive activity in a dose-independent manner and almost equivalent strength. As a comparison to MEMC, 5 mg/kg morphine also attenuated both phases of nociception while 100 mg/kg ASA only reduced nociception in the late phase. Overall, morphine was effective than ASA and MEMC in both phases of formalin test, while ASA was effective than MEMC in the late phase of the same test.

### 3.4. Capsaicin-Induced Paw Licking Test

**Figure 3:**
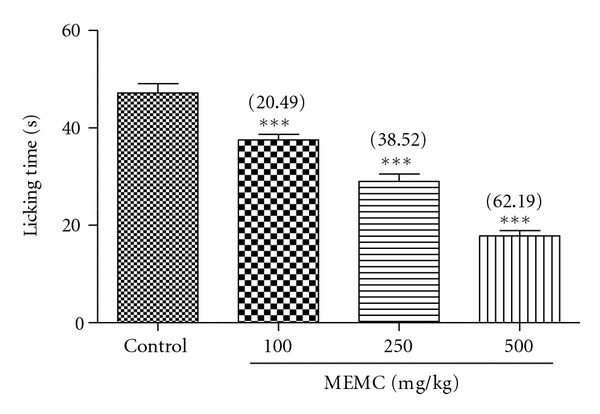
Effect of MEMC on capsaicin-induced paw licking test in rats. Each column represents the mean ± SEM of 6 rats. The rats were pretreated with vehicle (control, 10% DMSO) or MEMC (100, 250, and 500 mg/kg, p.o.) 60 min before injection of capsaicin (1.6 ug/paw, 20 *μ*L, i.pl.). The asterisks denote the significance levels as compared to control, ***P < 0.001 by one-way ANOVA followed by Dunnett's post hoc test.

The antinociceptive profile of MEMC assessed using capsaicin-induced paw licking test is shown in Figure 3. All doses of MEMC demonstrated a dose-dependent inhibition of capsaicin-induced nociception with percentage of analgesia ranging between 20 and 62%.

### 3.5. Glutamate-Induced Paw Licking Test

**Figure 4:**
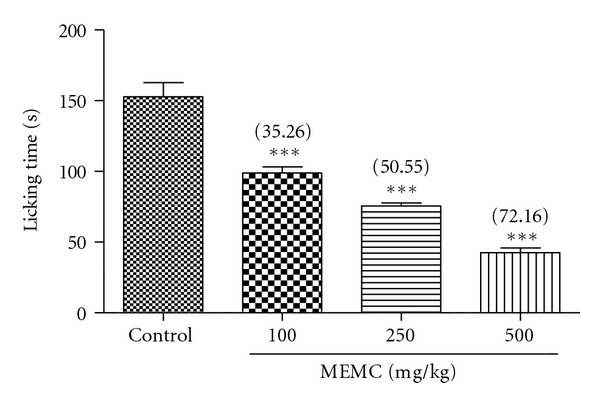
Effect of MEMC on glutamate-induced paw licking test in rats. Each column represents the mean ± SEM of 6 rats. The rats were pretreated with vehicle (control, 10% DMSO) or MEMC (100, 250, and 500 mg/kg, p.o.) 60 min before injection of glutamate (10 umol/paw, 20 *μ*L, i.pl.). The asterisks denote the significance levels as compared to control, ***P < 0.001 by one-way ANOVA followed by Dunnett's post hoc test.

Figure 4 shows the antinociceptive profile of MEMC against glutamate-induced paw licking test. All doses of MEMC also exerted a dose-dependent inhibition with the percentage of analgesia ranging from 35 to 72%.

### 3.6. Involvement of Opioid Receptors

The effect of nonselective opioid antagonist (5 mg/kg naloxone) on MEMC antinociceptive activity assessed using the abdominal constriction test, hot plate test, and formalin-induced paw licking test are shown in Figure 1, Table 1, and Figures 2(a) and 2(b), respectively. Interestingly, the 500 mg/kg MEMC antinociceptive activity was significantly (*P* < 0.05) inhibited in all tests. Moreover, naloxone reversed the extract antinociception in both phases of the latter test.

### 3.7. Involvement of NO/cGMP Pathway

**Figure 5:**
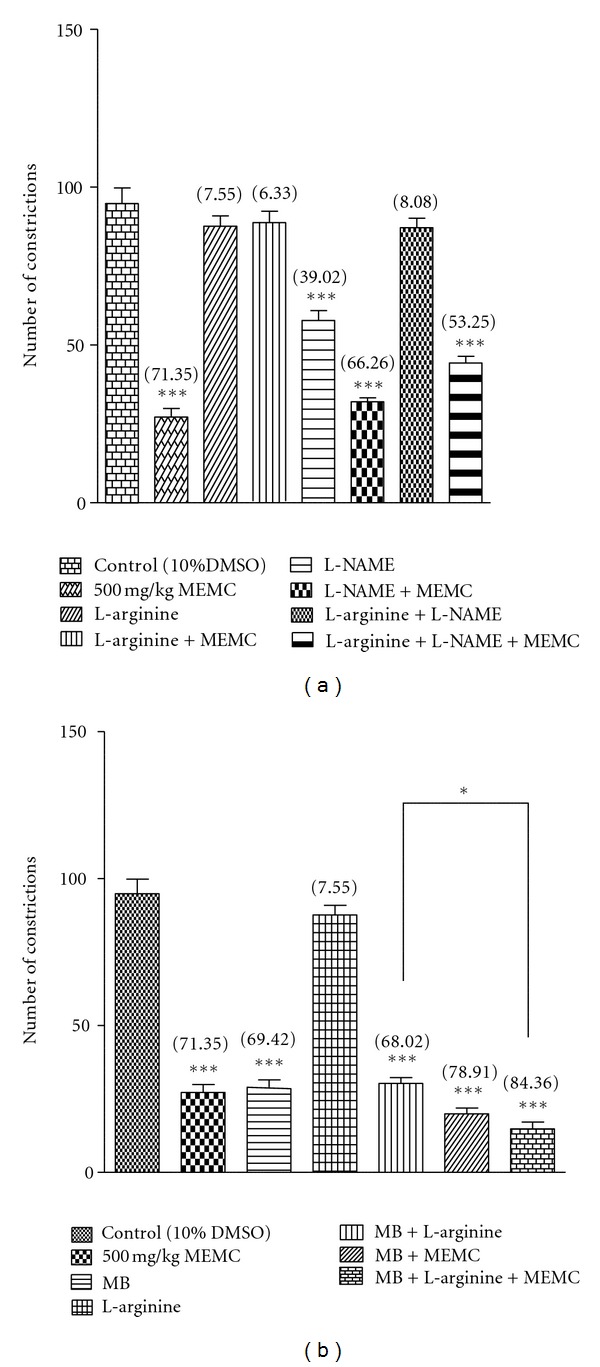
(a) Effects of L-arginine, L-NAME, and their combination on MEMC antinociception as assessed by acetic-acid-induced abdominal constriction test. The asterisks denote the significance levels as compared to control, ***P < 0.001 by one-way ANOVA followed by Dunnett's post hoc test. (b) Effects of L-arginine, methyline blue, and their combination on MEMC antinociception as assessed by acetic-acid-induced abdominal constriction test. The asterisks denote the significance levels as compared to control, ***P < 0.001 by one-way ANOVA followed by Dunnett's post hoc test. ***P < 0.001 by one-way ANOVA followed by Dunnett's post hoc test.

Figure 5(a) shows the effect of L-arginine, L-NAME, or their combination on 500 mg/kg antinociception assessed using the abdominal constriction test. L-arginine alone did not affect the acetic acid-induced nociception but significantly (*P* < 0.05) reversed the MEMC antinociceptive activity. On the other hand, L-NAME alone exerted significant (*P* < 0.05) antinociceptive activity and maintained the MEMC-induced antinociception as seen when the extract was given alone. L-arginine was also found to completely reverse the L-NAME-induced antinociception but when these compounds were combined and given together with the MEMC, the extract antinociceptive activity was maintained despite significant (*P* < 0.05) reduction in the percentage of analgesia recorded.

In additional study, MB alone exhibited significant (*P* < 0.001) antinociceptive activity and when given together with 500 mg/kg MEMC maintained the extract antinociceptive activity as seen when the extract was given alone (Figure 5(b)). Furthermore, L-arginine failed to reverse MB antinociceptive activity, whereas their combination also failed to inhibit the extract antinociceptive activity.

## 4. Discussion

The present study reported for the first time the antinociceptive potential of MEMC after an oral administration when assessed using the chemicals (acetic acid and formalin) and thermal stimuli models of nociception. The extract exhibited antinociceptive activity in both the chemicals (i.e., abdominal constriction test) and thermal (i.e., hot plate test) nociception models tested indicating that the extract possessed peripheral and central antinociceptive mechanisms, which is the characteristic of opioid analgesics (i.e., morphine). The involvement of both levels of antinociceptive mechanisms was further proven by the ability of MEMC to reverse the early and late phases of formalin test, which is also the characteristic of morphine. Other than that, the MEMC antinociception was demonstrated to involve modulation via the opioid receptors, and NO/cGMP pathway and inhibition of the vanilloid receptors and glutamate pathways. Interestingly, the involvement of opioid receptors in MEMC antinociception is postulated to take place at the peripheral and central levels as indicated by the ability of naloxone, a nonselective opioid antagonist, to attenuate the antinociceptive activity of MEMC in the abdominal constriction test and both phases of the formalin test.

The acetic-acid-induced abdominal constriction test, described as a typical model for inflammatory pain, has long been widely used as a tool to screen for analgesic or anti-inflammatory properties of new agents [17, 18] and, in most cases, used as a model to study the peripheral antinociceptive effect of extracts/compounds. This model of nociception is suggested to represent the stimulation of peripheral mechanism since the administration of phlogogen lead to an increase in the levels of cyclooxygenase (COX) and lipooxygenase (LOX) [19] and indirectly leads to the release of endogenous nociceptive mediators (e.g., prostanoids of the PGE_2_ and PGF_2*α*_ types, serotonin, histamine, cytokines, and eicosanoids) as well as other LOX products in peritoneal fluids that can induce various peripheral nociceptive neurons sensitive to NSAIDs within the peritoneal cavity [17, 20–22]. Prolong irritation of the peritoneal cavity has been associated with increase in the PGEs levels in the peritoneal fluid, which enhances capillary permeability [23, 24] and the release of glutamate and substance P from peripheral afferent fiber terminals [25]. Based on the above-mentioned fact, the ability of MEMC to attenuate the acetic-acid-induced abdominal constriction test suggests that the extract's antinociceptive mechanism involves, in part, its ability to inhibit COX and LOX in the peripheral tissues leading to decrease in PGEs synthesis and impediment of the pain transduction in primary afferent nociceptor. Interestingly, the abdominal constriction test is considered to be a very sensitive nociceptive model since it can detect antinociceptive effect of compounds/dose levels even at the lowest dosages that might not be effective in other tests (i.e., hot plate or tail-flick test) due to direct interaction of the extracts/compounds with the various peripheral receptors within the peritoneal cavity [26, 27]. However, this test also has been regarded as a nonspecific test as it could not be used to stipulate the involvement of peripheral or central mechanisms in the MEMC antinociception [28]. Furthermore, this model also has been considered to have poor specificity because certain drugs, such as muscle relaxants, and can also reduce the number of abdominal constriction [29]. Thus, further studies using other nociceptive models are required before a final conclusion on the mechanisms of antinociception of MEMC or other antinociceptive agents could be made.

In an attempt to determine whether the MEMC attenuated either the peripheral or central, or both levels of nociception, the thermal-induced nociceptive model (e.g., hot plate test) was performed. This model of nociception, which is predominantly a spinal reflex, is thought to involve supraspinal nociceptive processing and to be selective for centrally (opioids), but not peripherally (NSAIDs), acting analgesic compounds [8, 29–31]. According to Katzung [32], centrally acting drugs activate the release of endogenous peptide via the periaqueductal gray matter (PAG), which is then carried to the spinal cord to inhibit the pain impulse transmission within the dorsal horn. Based on the ability of highest dose of MEMC to prolong the latency to feeling discomfort, we suggest that the extract possessed mild centrally mediated antinociceptive activity against the thermal-induced nociception. The mild activity could be due to fact that the MEMC is a crude extract, which contained various types of bioactive phytochemicals, compared to morphine.

Another model of nociception that has been widely used to further support the antinociceptive effect observed in any new compounds is the formalin-induced paw licking test or formalin test [33]. Formalin injection into the rat's paw causes an immediate and intense increase in the spontaneous activity of C fiber afferent and evokes a distinct quantifiable behavior indicative of pain (i.e., licking of the injected paw) [34]. This test, which represents a model of persistent pain, can also be used to determine the ability of new compounds to affect peripheral or central nociceptive pathways due to its biphasic nociceptive characteristics, known as the early phase and late phase, resulting from the formalin administration [35]. The early phase, classified as a neurogenic pain, is an acute response observed immediately after the administration of formalin and persists for 5 min (0–5 min) as a result of a direct action of injected formalin on nociceptors. The late phase, classified as an inflammatory pain, is a tonic response resulting from the inflammatory processes generated by the release of inflammatory mediators such as histamine, serotonin, PGE and bradykinin [36], and activation of the neurons in the dorsal horns of the spinal cord [37, 38]. The late phase appears between 15 and 60 min (15–60 min) after the formalin administration. Based on the biphasic phases, the formalin test can also be used to determine the ability of new compounds to affect the noninflammatory (early phase) or inflammatory (late phase) associated nociceptive response. Centrally acting drugs (e.g., opioids) inhibit both phases, while peripherally acting drugs (e.g., NSAIDs) inhibit only the late phase. Based on the results obtained, the MEMC inhibited both phases of the formalin-induced nociception suggesting its ability to act at central nociceptive level, which is the characteristic of morphine. This finding further confirms the MEMC centrally mediated antinociceptive characteristic observed using the hot plate test. Moreover, ability to attenuate the late phase implies that the extract possesses not only antinociceptive, but also anti-inflammatory activity [8]. Overall, results from the three assays suggested that MEMC contains bioactive compound(s) with central and peripheral antinociceptive actions and additional anti-inflammatory activity.

Other than the role of opioid receptors, which has been proven and discussed earlier, further studies were also carried out to study the effect of MEMC against vanilloid receptors induced nociceptive transmission and to explore the role of glutamatergic system and NO/cGMP pathway in the modulation of MEMC antinociception. In an attempt to study the effect of MEMC against nociceptive transmission via vanilloid receptors, the extract was assayed against capsaicin-induced paw licking test. Capsaicin, an active ingredient in hot chili peppers, directly stimulates vanilloid receptor 1 or transient receptor potential cation channel subfamily V member 1 (TRPV1) [39]. This types of receptors, which are involved in the transmission and modulation of nociceptive activity, as well as the integration of diverse painful stimuli, selectively acting on unmyelinated C-fibers and thinly myelinated A primary sensory neurones within the peripheral nervous system (PNS), as well as tissues within the central nervous system (CNS) [40–43]. Interestingly, antagonists of TRPV1 receptors have been reported to exhibit a pain-relieving activity [44] and were effective in reducing nociception from inflammatory as well as neuropathic pain models in rats [45]. Based on our finding, the oral administration of MEMC produced a neurogenic inhibition against capsaicin-induced nociception in a dose-dependent manner indicating the ability of MEMC to inhibit nociceptive transmission initiated by TRPV1 activation. As the TRPV1 receptors are also triggered by heat and could be involved in the thermal-induced nociception (e.g., hot plate test) [46], the present findings seem to suggest the potential of MEMC as an antagonist of TRPV1 receptors at the peripheral and central levels.

In another attempt to determine the role of glutamatergic system in the modulation of MEMC antinociception, the extract was subjected to the glutamate-induced paw licking test. Glutamate is a major excitatory neurotransmitter in the CNS [47], and various reports have shown that the glutamate and glutamatergic receptors (both ionotropic and metabotropic glutamate receptors) are important in the peripheral, spinal, and supraspinal nociceptive neurotransmission [48–50], which is greatly mediated by both N-methyl-D-aspartate (NMDA) and non-NMDA receptors, as well as by the release of NO and NO-related substances [51]. On the other hand, NMDA receptor antagonists have been proven to inhibit the spread of pain sensation and to reduce the hyperexcitability of spinal cord neurons triggered by C-fiber stimulation [52, 53]. In addition, activation of glutamate receptors also have been reported to contribute to the maintenance of peripheral nociceptive processes that are associated with inflammatory, but not physiological pain [54], which is concurrent with report that administration of glutamate receptor antagonist inhibited the inflammatory, but not neurogenic phases of the formalin test [55]. Based on our findings, glutamatergic system did involve in the modulation of MEMC antinociception.

In an attempt to determine the role of L-arginine/NO/cGMP pathway in mediating the MEMC antinociception, the extract's antinociceptive activity was prechallenged with L-arginine (a NO donor), L-NAME (an inhibitor of NOS), and MB (an inhibitor of cGMP pathway) followed by subjection to the abdominal constriction test. NO is a biological molecule found inside and between cells that reactively acts as a mediator to convey biochemical signals resulting in a wide spectrum of effects on different biological systems, including the CNS [56] and PNS [57]. The results of NO production are stimulation of soluble guanylate cyclase (sGC) and rise in the cGMP level within the target cells [57]. NO has been reported to modulate pain mechanism at both the PNS and CNS levels [58] with the high level of NO induces pain and vice versa [59]. Other than that, NO has been implicated as a mediator or modulator in analgesic drug function [60]. In line with those reports, the peripheral activation of the NO-cGMP pathway has been implicated in various nociceptive conditions [61]. The differential effect that NO has might be due to the fact that each tissue might be or is predominantly innervated by different subsets of primary nociceptive neurons [62]. Based on our findings, increase in NO level reversed the MEMC antinociception while reduction in NO level did not affect the extract antinociception. This observation is concurrent with suggestion that the effect of NO depends on dosage levels and the rate and timing of its release [58, 59]. In addition, failure of L-NAME to enhance but instead maintain MEMC antinociception is suggested to be due to the amount of NO inhibited within the peripheral level was enough to prevent activation of various nociceptive pathways associated with NO (i.e., COX, glutamatergic, or TRPV1 systems). Furthermore, based on our observation, it is suggested that the inhibition of cGMP pathway, whether in the presence or absence of NO, will lead to antinociception and, in the presence of MEMC/other antinociceptive agents, will enhance the agents antinociceptive effect. MB has been widely applied in researches involving pain perception because it has been shown to act as a less specific and potent guanyl cyclase (GC) inhibitor, which directly blocked NOS and decreased the accumulation of cGMP. GC is one of the main targets of NO [63], thus, MB is frequently used to clarify the involvement of cGMP pathway in the effects of NO system on mechanisms of pain. The ability of MB to enhance MEMC antinociceptive activity corroborates with previous reports [59, 61–63]. Thus, the present findings supported the involvement of NO/cGMP pathway in the modulation of peripheral antinociception of MEMC.

We have recently reported the presence of flavonoids, tannins, and saponins in the MEMC [11]. Flavonoids could be responsible for the observed antinociceptive activity of MEMC as this class of compounds has been reported to modulate pro-inflammatory gene expression like inducible NOS and COX-2 [64]. Thus, the ability of flavonoids to modulate various pain pathways could be used to explain on the recent observations.

## 5. Conclusion

The present study demonstrated that the MEMC possessed both central and peripheral antinociceptive activities that involve inhibition of COX activity or PGE synthesis as well as activation of opioid, glutamatergic system, and NO/cGMP pathway. The MEMC also exhibited inhibitory effect against TRPV1-receptor-mediated nociceptive transmission. These activities are attributed to the possibly synergistic action of flavonoids, saponins, and tannins present in the extract. Further studies are now in progress to determine the bioactive compound(s) responsible for the analgesic properties of *M. calabura*.

## Figures and Tables

**Table 1:** Effect of MEMC on the hot plate test in mice.

Treatment	Dose (mg/kg)	Latency of discomfort(s) at respective time interval (min)						
		0 min	60 min	90 min	120 min	150 min	180 min	210 min
10% DMSO		6.97 ± 0.22	6.90 ± 0.23	6.15 ± 0.15	6.72 ± 0.16	6.92 ± 0.23	6.88 ± 0.29	6.35 ± 0.17

Morphine	**5**	5.77 ± 0.15	17.37 ± 1.03^a^	18.25 ± 0.74^a^	16.52 ± 1.22^a^	13.67 ± 1.43^a^	11.22 ± 1.11^a^	10.48 ± 0.58^a^

MEMC	**100**	6.50 ± 0.13	7.12 ± 0.17	6.40 ± 0.32	6.58 ± 0.32	6.87 ± 0.24	6.97 ± 0.50	6.75 ± 0.26
	**250**	5.68 ± 0.14	6.93 ± 0.16	7.50 ± 0.48^a^	7.75 ± 0.36	7.68 ± 0.37	7.43 ± 0.24	7.72 ± 0.40^a^
	**500**	6.95 ± 0.18	10.65 ± 0.47^ab^	10.22 ± 0.42^ab^	9.80 ± 0.95^ab^	9.48 ± 0.38^ab^	9.88 ± 0.18^ab^	9.28 ± 0.32^a^

Naloxone	**5**	6.38 ± 0.27	6.43 ± 0.41	5.98 ± 0.46	6.10 ± 0.21	5.93 ± 0.68	6.13 ± 0.58	5.67 ± 0.54

Naloxone + MEMC	**5 + 500**	6.02 ± 0.27	5.75 ± 0.23^c^	5.55 ± 0.25^c^	5.65 ± 0.47^c^	5.75 ± 0.64^c^	5.97 ± 0.61^c^	5.75 ± 0.81^c^

^a^Data differed significantly (*P* < 0.05) when compared against the control group.

^b^Data differed significantly (*P* < 0.05) when compared against the 5 mg/kg morphine-treated group.

^c^Data differed significantly (*P* < 0.05) when compared against the 500 mg/kg MEMC-treated group.
